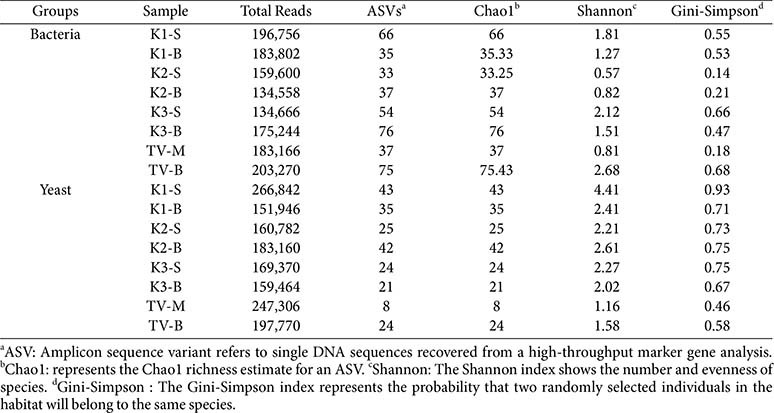# Comparative Analysis of Microbial Communities and Biopolymer Production in Kombucha

**DOI:** 10.4014/jmb.2508.08004

**Published:** 2025-10-28

**Authors:** Younhee Nam, Gayeon Seo, Younghoon Kim, Soo Rin Kim, Jong Nam Kim

**Affiliations:** 1 Department of Advanced Bioconvergence, Kyungpook National University, Daegu 41566, Republic of Korea; 2 Department of Food Science and Nutrition, Dongseo University, Busan 48513, Republic of Korea; 3 Department of Agricultural Biotechnology and Research Institute of Agriculture and Life Science, Seoul National University, Seoul 08826, Republic of Korea; 4 School of Food Science and Biotechnology, Kyungpook National University, Daegu 41566, Republic of Korea

**Keywords:** Kombucha, SCOBY, BC, microbiome, NGS

## Abstract

While the microbial diversity of kombucha has been previously investigated, only a limited number of studies have explicitly distinguished between the symbiotic culture of bacteria and yeast (SCOBY) and the liquid broth, and even fewer have directly associated microbial diversity with bacterial cellulose production. This study investigated the microbial communities present in commercially available kombucha products by using both culture based and molecular analysis methods, along with metabolite profiling by chemical analyses. Culture based methods identified key cellulose-producing strains, including *Komagataeibacter intermedius*, *K. rhaeticus*, and *Novacetimonas hansenii*, while next-generation sequencing revealed *Komagataeibacter* as the dominant bacterial genus in kombucha. Yeast communities in kombucha were predominated by *Zygosaccharomyces bisporus* and *Z. parabailii*. As fermentation progressed, all kombucha samples exhibited typical fermentation dynamics, characterized by progressive sucrose depletion and an increase in ethanol and acetate production. Given the promising industrial applications of bacterial cellulose, the biopolymer content of kombucha was evaluated. Among the kombucha samples, K2 showed the highest cellulose yield (4.50 ± 2.28 g), and *N. hansenii* was identified as the most efficient cellulose producer among the isolates. This integrative approach provides critical insights into the role of microbial communities in regulating kombucha fermentation. Specifically, this study delineated the core microbiota required for stable fermentation and identified strains with enhanced cellulose producing capacity. Beyond defining the key microbial taxa associated with kombucha production, these findings underscore the industrial potential of kombucha derived cellulose producers and present a strategy for optimizing bacterial cellulose yield in large scale applications.

## Introduction

Kombucha, a naturally carbonated beverage originating from China, is produced using a sweetened tea infusion with a symbiotic culture of bacteria and yeast (SCOBY) under aerobic conditions. The fermentation process of kombucha is generally divided into two stages: primary and secondary fermentation. The primary fermentation stage typically lasts for 14 to 21 days and involves microbial proliferation and SCOBY formation. The secondary fermentation stage extends for 2 to 7 days after primary fermentation and involves the addition of flavoring agents such as fruits, which further enhance the organoleptic properties of the beverage [1, 2].

During fermentation, a variety of bioactive compounds are formed in kombucha, including organic acids, amino acids, vitamins, probiotics, polyphenols, and antioxidants. These components possess several health-promoting properties, such as anti-inflammatory, antimicrobial, antidiabetic, and antioxidant effects [2, 3]. Because of the increasing consumer demand for functional beverages, the market for kombucha products has expanded rapidly over the past two decades, with increased availability of these products across both online and offline retail platforms [4].

Kombucha fermentation is initiated with the inoculation of SCOBY, which comprises various yeast species and acetic acid bacteria (AAB). Several genera of yeast, such as *Zygosaccharomyces*, *Candida*, *Brettanomyces*, *Schizosaccharomyces*, and *Saccharomyces*, hydrolyze sucrose into glucose and fructose, which are subsequently converted into ethanol. AAB, including *Komagataeibacter* and *Gluconobacter*, oxidize ethanol to produce acetic acid and CO_2_, which contribute to acidity, tanginess, and carbonation of the final product [5, 6]. Several studies have also reported the presence of lactic acid bacteria (LAB), such as *Lactobacillus* and *Lactococcus*, in kombucha fermentation [7].

Among AAB, *Komagataeibacter xylinus* (formerly known as *Acetobacter xylinum* or *Gluconacetobacter xylinus*) is known for its high capacity to produce bacterial cellulose (BC), which plays a crucial role in pellicle (SCOBY) formation. AAB belongs to the family *Acetobacteraceae*, which comprises diverse genera, including *Komagataeibacter* and *Gluconobacter* [8]. Unlike plant derived cellulose, BC is devoid of lignin, hemicellulose, and pectin, resulting in exceptional purity and material properties such as high water holding capacity, biocompatibility, biodegradability, and mechanical strength. These characteristics render BC highly valuable for applications in biomedical engineering, food packaging, and tissue scaffolds [9].

Although kombucha is traditionally produced using undefined microbial consortia, industrial scale production of kombucha requires the use of well characterized and safe microbial strains to ensure the reproducibility, safety, and high quality of the final kombucha products [10]. These challenges are also encountered in the production of BC, which is primarily synthesized by AAB in the SCOBY. Although specific species such as *Komagataeibacter xylinus* are known to be efficient BC producers, the overall contribution of individual microorganisms in mixed cultures remains poorly understood [8]. Therefore, the isolation of pure microbial strains is essential for accurate taxonomic and functional analysis [11]. While extensive research has employed culture dependent methodologies and high throughput sequencing technologies to characterize the microbial ecology of kombucha fermentation systems, limited studies have systematically differentiated the microbial consortia within the symbiotic culture of bacteria and yeast (SCOBY) biofilm from those in the fermentation broth or established direct correlations between these distinct community architectures and their metabolic outputs. This investigation integrates next-generation sequencing (NGS), culture dependent isolation techniques, comprehensive metabolomics profiling, and bacterial cellulose (BC) quantification to elucidate these microbiome metabolome relationships, providing critical insights for targeted strain selection and process optimization in commercial scale kombucha production.

This study characterized the microbial community structure of kombucha fermentation systems through integrated next-generation sequencing (NGS) and culture dependent isolation approaches. Comprehensive metabolomic and biopolymer analyses were conducted to profile bioactive compounds and extracellular matrix components. This multi omics strategy aimed to identify the core microbiome of kombucha and establish correlations between microbial consortium dynamics and bacterial cellulose (BC) biosynthesis. Consequently, this investigation provides fundamental insights into kombucha microbial ecology and metabolic pathways while exploring the biotechnological potential of bacterial cellulose, a high value biopolymer with diverse industrial applications in food, biomedicine, and materials engineering

## Materials and Methods

### Sample Preparation

Three commercial kombucha samples (K1, K2, and K3) were prepared for microbial and chemical analyses, and traditional vinegar (TV) was included as a control because of its low pH and the presence of AAB.

To obtain 1 L of kombucha, a green tea infusion was prepared by mixing 4.8 g of green tea leaves (*Camellia sinensis*) with 700 ml of distilled water and brewing the mixture at 95°C for 5 min. After removing the tea leaves, 100 g of sucrose (Junsei Chemical Co., Ltd., Japan) was added, and the mixture was sterilized by autoclaving. The sterilized tea solution was cooled to room temperature, and 150 g of SCOBY and 200 ml of previously fermented kombucha broth were subsequently inoculated. All kombucha samples were incubated aerobically at room temperature for 21 days. Broth samples were collected at 7 day intervals for chemical analysis, and both SCOBY and broth samples were used for microbial analysis at the end of the fermentation period.

TV was prepared using commercially available, unfiltered Korean traditional rice wine. The wine was transferred into sterilized glass containers covered with sterile cheesecloth to allow aerobic fermentation and incubated at 25°C with daily stirring to ensure thorough mixing of oxygen with the liquid. Vinegar samples, including both broth and membrane fractions, were collected on day 21 for microbial analysis. All experiments were performed in triplicate to ensure reproducibility and data reliability.

### Isolation and Identification of Bacteria and Yeast

To isolate bacteria, yeast, and LAB, two types of samples were collected at different stages. For kombucha, the cellulose biofilm (SCOBY, designated as S) and the kombucha broth (designated as B) were used. For TV, the pellicle at the liquid air interface (designated as M) and the vinegar broth (designated as B) were collected. Selective and modified media were used for microbial isolation based on previously established protocols.

Different selective media were used for microbial isolation, including glucose yeast extract (GY) agar (glucose 20 g/l, yeast extract 10 g/l, agar 20 g/l) for acetic acid bacteria (AAB), malt extract agar (MEA; BD Difco, USA) for yeasts, nutrient agar (BD Difco) and Lactobacilli MRS agar (BD Difco) for *Lactobacillus* species, and LGI agar (0.06% KH_2_PO_4_, 0.02% K_2_HPO_4_, 0.02% MgSO_4_, 0.002% CaCl_2_, 0.001% FeCl_3_, 0.0002% Na_2_MoO_4_, and 10% sucrose) for nitrogen fixing bacteria [12].

A 1 μl aliquot of each sample was streaked onto the plates of respective media using a sterile inoculation loop. The plates were incubated for 3 to 5 days at 25°C for GY and nutrient agar, 30°C for MEA and LGI agar, and 37°C for MRS agar.

The isolates were preliminarily identified using a Gram staining kit (YD Diagnostics, Yongin, Republic of Korea). Morphological characteristics were examined with a light microscope (Model YS2-H, Nikon, Japan) at 1,000× magnification under an oil immersion lens. All isolated strains were preserved as 50% glycerol stocks and stored at -80°C for further analysis.

Molecular identification was performed by Macrogen (Republic of Korea). For bacterial isolates, 16S rRNA gene sequencing was conducted using primers 785F (5'-GGATTAGATACCCTGGTA-3') and 907R (5'-CCGTCAATTCMTTTRAGTTT-3') [13, 14]. The obtained sequences were compared against the NCBI rRNA database to reveal taxonomic identity.

### Metagenomic Analysis

Metagenomic DNA was extracted using the DNeasy Blood & Tissue Kit (Qiagen, Germany), in accordance with the manufacturer’s protocol with slight modifications. For broth samples, 5 ml of each sample was centrifuged at 12,000 rpm for 3 min, and the supernatant was discarded. The resulting pellet was washed with 500 μl of 0.8% (w/v) NaCl solution and centrifuged again under the same conditions.

For SCOBY samples, the cellulose pellicle was cut into approximately 3 × 3 mm pieces and washed twice with 500 μl of 0.8% (w/v) NaCl. After each wash, the samples were centrifuged at 12,000 rpm for 3 min, and the supernatant was discarded.

Following the washing steps, both broth and SCOBY pellets were subjected to DNA extraction in accordance with the protocol provided by the kit manufacturer. The concentration and purity of the extracted DNA were measured using a NanoDrop spectrophotometer (NanoDrop OneC, Thermo Fisher Scientific, USA). NGS for the extracted DNA was performed by Macrogen.

For bacterial community analysis, the V3–V4 region of the 16S rRNA gene was amplified using primer pair 341F (5'-CCTACGGGNGGCWGCAG-3') and 805R (5'-GACTACHVGGGTATCTAATCC-3'). Yeast community profiling was conducted by targeting the ITS3–ITS4 region using primers ITS3 (5'-GCATCGATGAAGAACGCAGC-3') and ITS4 (5'-TCCTCCGCTTATTGATATGC-3').

The following PCR protocol was used: initial denaturation at 95°C for 3 min, followed by 25 cycles of denaturation at 95°C for 30 sec, annealing at 55°C for 30 sec, and extension at 72°C for 30 sec, with a final extension step at 72°C for 5 min. The amplified products were used to construct sequencing libraries with Herculase II Fusion DNA Polymerase and the Nextera XT Index Kit v2 (Illumina, USA). Sequencing was performed on the Illumina MiSeq system in accordance with the manufacturer’s instructions.

Bioinformatics analysis was conducted using QIIME (v1.9.0), DADA2 (v1.18.0), MAFFT (v7.475), and FastTreeMP (v2.1.10).

### Metabolite Analysis

Three types of kombucha broth were centrifuged at 4,500 ×*g* for 10 min, and the resulting supernatants were filtered through 13 mm syringe filters equipped with hydrophilic PTFE membranes and glass fiber prefilters (pore size: 0.2 μm; DOUBLE, Republic of Korea) for subsequent analyses.

The pH of the filtered supernatants was measured using a pH meter (Starter 300, OHAUS, USA). Concentrations of sucrose, glucose, fructose, lactic acid, acetic acid, and ethanol were determined by high performance liquid chromatography (1260 Series, Agilent Technologies, USA). Chromatographic separation was achieved using a Rezex ROA-Organic Acid H^+^ (8%) column (150 mm × 4.6 mm; Phenomenex Inc., USA), equipped with a refractive index detector. The mobile phase was 0.005 N H_2_SO_4_, delivered at a constant flow rate of 0.6 ml/min, and the column temperature was maintained at 50°C throughout the analysis.

### Biopolymer Analysis

The cellulose content produced by bacterial strains isolated from kombucha samples was quantified. The bacterial strains were precultured in GY broth for 24–48 h and subsequently cultured for 21 days under the same conditions as those used for kombucha fermentation. BC was quantified using a previously reported method [15], with a glucose standard for calibration.

### Statistical Analysis

All experiments were conducted in biological triplicate, and data are presented as mean values. Standard deviations (SD) were calculated to assess analytical variability in metabolite and biopolymer quantification assays. Statistical differences between experimental groups were evaluated using one-way analysis of variance (ANOVA) with IBM SPSS Statistics software (version 29.0; IBM Corp., USA). Multiple pairwise comparisons were performed using Scheffé’s post hoc procedure, with statistical significance set at *p* < 0.05.

## Results and Discussion

### Isolation and Identification of Bacterial and Yeast Species from Kombucha

To characterize the cultivable microbial diversity within kombucha fermentation systems, the taxonomic distribution of bacterial and fungal communities was analyzed across experimental kombucha samples (K1, K2, and K3) and benchmarked against the traditional vinegar (TV) control sample. Culture dependent isolation coupled with molecular identification through 16S rRNA gene and internal transcribed spacer (ITS) region sequencing yielded 13 distinct microbial taxa, comprising 8 bacterial species and 5 yeast species (Table 1). Notably, microbial composition exhibited clear variations depending on the sample fraction, with SCOBY (S) or membrane (M) showing distinct profiles compared to the broth (B), as well as among different kombucha batches.

Among bacterial isolates, *Gluconobacter oxydans* was the most frequently detected species. This species was identified in the broth of all three kombucha samples but was absent in both SCOBY and TV samples. *Komagataeibacter intermedius* and *K. swingsii* were exclusively isolated from the K1 sample, and *K. intermedius* was present in both SCOBY and broth fractions, whereas *K. swingsii* was detected only in the broth. *K. rhaeticus* was identified in both the SCOBY of K2 (K2-S) and the broth of K3 (K3-B), suggesting its occurrence across multiple kombucha batches.

Among the culturable bacterial populations, *Gluconobacter oxydans* emerged as the predominant acetic acid bacterium, exhibiting universal presence in the fermentation broth across all three kombucha samples while remaining undetectable in both SCOBY biofilm matrices and the TV control. Two *Komagataeibacter* species demonstrated sample specific distribution patterns within the K1 sample: *K. intermedius* exhibited dual compartment colonization across both SCOBY and broth fractions, whereas *K. swingsii* displayed strict broth phase localization. Notably, *K. rhaeticus* demonstrated inter batch distribution heterogeneity, with detection in the SCOBY biofilm of K2 (K2-S) and the liquid broth of K3 (K3-B), indicating cross-batch ecological adaptability and niche specific colonization patterns.

Additional bacterial species, including *Margalitia shackletonii*, *Acetobacter ghanensis*, and *Lacticaseibacillus paracasei*, were exclusively isolated from TV samples. *M. shackletonii* and *A. ghanensis* were identified in the membrane (TV-M) and broth (TV-B) fractions, with *M. shackletonii* present in both fractions. *L. paracasei* was also detected in both fractions of TV. Notably, *Bacillus amyloliquefaciens* and *B. halotolerans* were exclusively detected in TV-B, suggesting that these spore-forming taxa may be uniquely associated with the vinegar fermentation environment.

Yeast species exhibited more restricted distribution patterns compared to bacterial taxa. *Brettanomyces bruxellensis* and *Dekkera bruxellensis* were detected in the SCOBY of the K1 sample, with the former species additionally identified in the broth of K1, suggesting its persistence across both fractions. *Zygosaccharomyces parabailii* and *Z. bisporus* were primarily detected in K2 and K3 samples. *Z. bisporus* was found in both K2-S and K3-B, whereas *Z. parabailii* was detected exclusively in K1-B. These results highlight species level diversity among the yeast isolates of kombucha.

Overall, bacterial diversity was more pronounced in broth fractions than in SCOBY, particularly for *Gluconobacter* spp. and *Komagataeibacter* spp., which contribute significantly to acetic acid production. In contrast, the microbial composition of TV samples was distinct and dominated by *L. paracasei* and *Bacillus* spp., which were not detected in the kombucha samples.

Bacterial genera detected in kombucha samples included *Komagataeibacter*, *Gluconobacter*, *Novacetimonas*, and *Acetobacter*. *Komagataeibacter* spp. are characterized as obligate aerobic, gram negative, alpha proteobacteria and are classified as AAB, they are frequently isolated from vinegar, fruits, fruit juices, kombucha, etc. They produce both acetic acid and BC and exhibit strong resistance to acetic acid. *Gluconobacter* spp. and *Novacetimonas hansenii* also synthesize cellulose with properties such as high tensile strength, water holding capacity, crystallinity, and hydrophilicity, which support their application in diverse areas [16, 17].

*Acetobacter* spp. possess strong enzymatic activities that facilitate the conversion of ethanol and glucose into acetic and gluconic acids. Therefore, they are widely utilized in brewing processes. Additionally, *Acetobacter* spp. can oxidize various alcohols and sugars into a wide range of organic compounds [18].

Among *Bacillus* species, *Margalitia* is a recently proposed genus originating from the reclassification of certain *Bacillus* members [19, 20].

Yeast genera identified in kombucha samples included *Dekkera*, *Brettanomyces*, and *Zygosaccharomyces*. *D. bruxellensis* was identified as a spoilage yeast associated with wine fermentation. This species is a phylogenetically distant relative of *Saccharomyces cerevisiae*. It is also commonly detected in Belgian lambic beers, kombucha, and sourdough. This species is particularly adapted to harsh environmental conditions, including high ethanol concentrations, low pH, and limited nitrogen availability [21]. *B. bruxellensis* is another recognized spoilage yeast in wine and is also a prevalent species in a variety of alcoholic beverages. Moreover, it has industrial significance as one of the few yeast species that can utilize starch in continuous alcohol fermentation systems [22].

In contrast, the TV used as the control sample harbored several bacterial taxa, including *Acetobacter*, *Lacticaseibacillus*, *Margalitia*, and *Bacillus*. Among these, *L. paracasei* (formerly *Lactobacillus paracasei*), which is commonly isolated from artisanal fermented beverage starters, can confer various health promoting effects, including antimicrobial activities, immune response modulation, anti-inflammatory and antioxidant effects, and lipid metabolism enhancement [23].

*B. amyloliquefaciens* has been previously isolated from makgeolli and can inhibit the growth of pathogenic microorganisms. This species can synthesize antimicrobial lipopeptides, including iturin, fengycin, bacilysin, and surfactin [24]. *B. halotolerans* produces lipopeptides and exerts protective effects on plants against fungal pathogens [25].

### α- and β-Diversity of Microbial Communities in Kombucha Samples

NGS was conducted to assess microbial alpha (α) and beta (β) diversity within SCOBY biofilm matrices and kombucha samples. Metagenomic analysis generated substantial sequencing depth per sample (134,000 - 266,000 high quality reads), achieving complete taxonomic coverage as demonstrated by Good’s estimator values of 1.0 across all specimens. Microbial community diversity was quantified using established ecological indices, including species richness (Chao1 estimator), information entropy (Shannon index), and dominance (Gini-Simpson index), while community structure relationships were evaluated through principal coordinate analysis (PCoA) with phylogenetically weighted UniFrac distance matrices implemented in QIIME v1.9.0. Given the constraints of limited biological replication, all diversity analyses are presented as exploratory findings requiring validation through expanded experimental designs.

The number of bacterial amplicon sequence variants (ASVs) ranged from 33 to 76, with species richness (Chao1 index) exhibiting similar trends (Table 2). Notably, bacterial richness was higher in broth fractions (*e.g.*, K3-B with 76 ASVs) than in SCOBY fractions (*e.g.*, K2-S with 33 ASVs). This finding is consistent with previous reports of greater bacterial diversity in kombucha broths. TV samples exhibited varying richness, with TV-B displaying high species richness (Chao1 index = 75), comparable to that of kombucha broths.

Shannon diversity indices for the bacterial communities ranged from 0.57 to 2.68, with the highest diversity observed in K3-S and TV-B, and the lowest diversity in K2-S and TV-M. Gini-Simpson indices showed similar trends, suggesting relatively low bacterial diversity in some kombucha and vinegar fractions, where bacterial communities were dominated by a limited number of taxa. For eukaryotic communities, the ASV count and species richness were generally lower than those of bacterial communities, ranging from 8 to 42. Kombucha, SCOBY, and broth fractions consistently exhibited higher yeast species richness and species diversity than vinegar samples. Shannon and Gini-Simpson indices were the highest in kombucha samples, with K1-S demonstrating peak diversity (Shannon index = 4.41, Gini-Simpson index = 0.93).

**Fig. 1:**
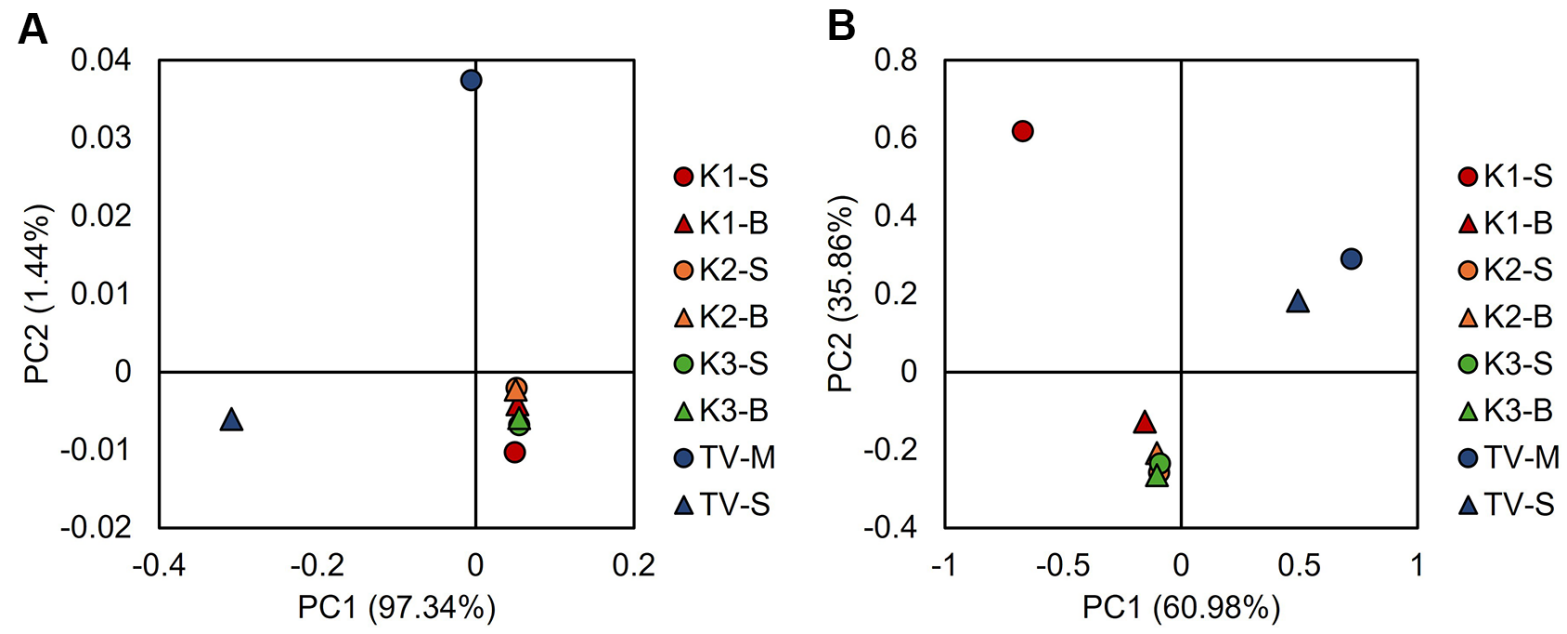
Principal Coordinates Analysis (PCoA) plot based on weighted UniFrac distance illustrating β-diversity among three kombucha samples and a traditional vinegar sample. (**A**) Bacteria, (**B**) Yeast.

Principal coordinate analysis (PCoA) based on phylogenetically weighted β-diversity metrics demonstrated clear taxonomic clustering patterns that distinguished kombucha samples from traditional vinegar (TV) control samples, revealing fundamental differences in microbial community architecture. For bacterial consortia, the first two principal components (PC1 and PC2) achieved complete sample segregation into distinct ecological clusters, with kombucha fractions exhibiting convergent community structures that were phylogenetically distinct from TV samples. Similarly, fungal community ordination revealed discrete clustering patterns for all samples except K1-S, TV-M, and TV-B, indicating substrate specific yeast population differentiation when analyzed across the primary ordination axes. TV samples consistently exhibited divergent microbial assemblages compared to kombucha fermentation systems (Fig. 1).

These ordination patterns reflect distinct ecological drivers governing microbial community assembly and succession. Kombucha samples, utilizing sweetened tea substrates and standardized SCOBY inoculum, exert selective pressure favoring tea polyphenol adapted, cellulose biosynthetic acetic acid bacteria (particularly *Komagataeibacter* species), while yeast populations undergo selective adaptation to polyphenolic stress gradients and osmotic fluctuations characteristic of tea based fermentation matrices [26]. Conversely, traditional vinegar production relies on spontaneous fermentation of cereal derived substrates, fostering indigenous microbial consortia dominated by *Acetobacter*, *Bacillus*, and lactic acid bacteria (LAB) populations, ultimately resulting in enhanced taxonomic diversity and metabolic pathway redundancy spanning lactic acid fermentation to acetogenesis [27].

Collectively, these findings demonstrate that kombucha harbors a more diverse and compositionally distinct microbial community than that observed in TV, and the tight clustering of kombucha samples in Principal Coordinates Analysis (PCoA) is consistent with a stable and reproducible microbial consortium across fermentation.

### Phylogenetic Analysis of Microbial Communities in Kombucha Samples

Phylogenetic relationships among the microbial communities of kombucha samples were assessed using genetic distance analysis. A dendrogram was constructed based on Nei’s genetic distance matrix to determine the genetic relationships among the samples. UPGMA clustering of bacterial communities clearly separated kombucha samples from TV fractions, with a low similarity value of 0.1769, indicating significant divergence from kombucha. Kombucha samples exhibited relatively high similarity in microbial communities, forming a closely related cluster, although K1-S appeared as a distinct subgroup.

**Fig. 2:**
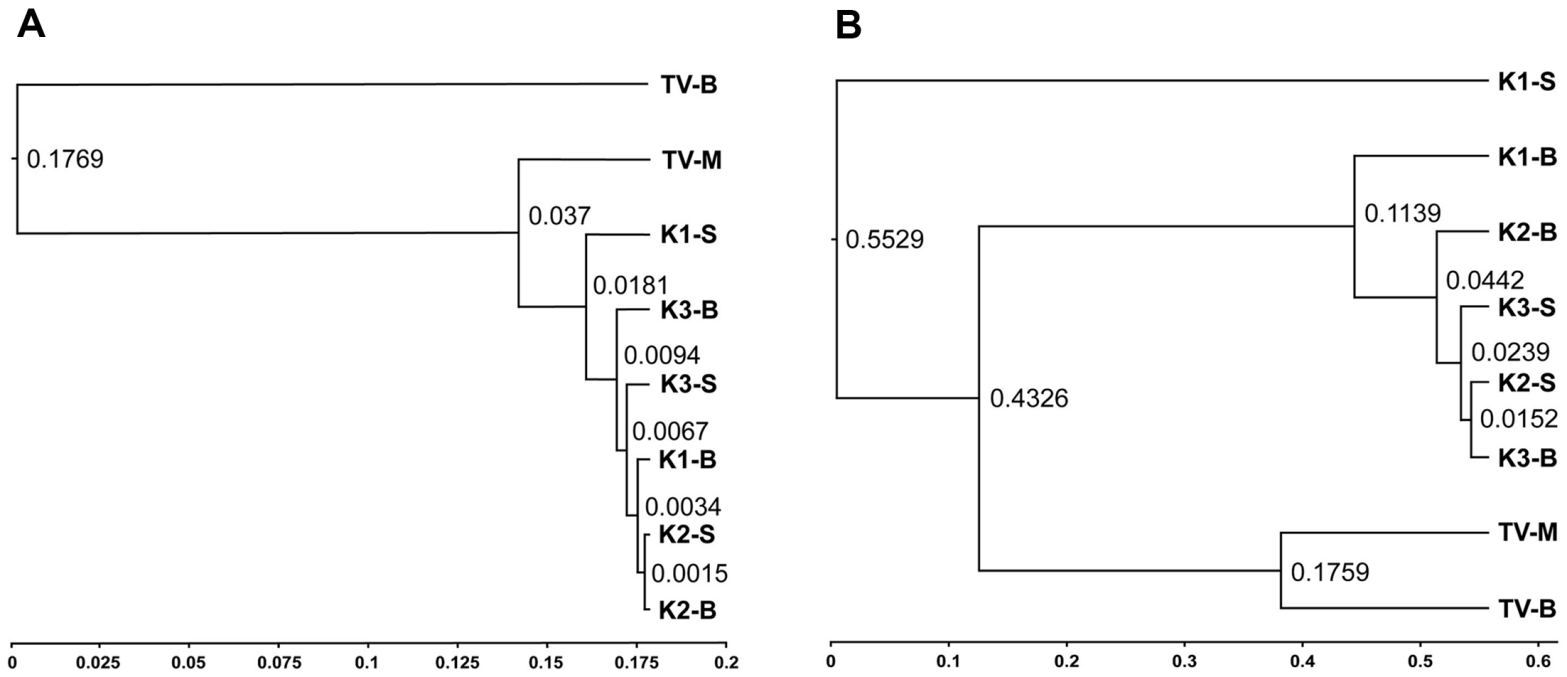
UPGMA phylogenetic tree based on weighted UniFrac distance showing phylogenetic relationships among the three kombucha samples and the traditional vinegar sample. (**A**) Bacteria, (**B**) Yeast.

A similar pattern was observed for yeast communities. TV-M and TV-B displayed a similarity of 0.1769, but remained substantially distant from kombucha samples, suggesting marked structural differences in the eukaryotic microbiota. Among the kombucha samples, K2 and K3 showed relatively high similarity, while K1 formed an independent cluster, indicating a distinct phylogenetic profile (Fig. 2).

### NGS Analysis of Bacterial and Yeast Communities in Kombucha Samples

To comprehensively characterize the bacterial and yeast microbial consortia within kombucha samples, high-quality genomic DNA was extracted from distinct SCOBY biofilm matrices and fermentation broth compartments, followed by targeted amplicon sequencing analysis. Given the inherent constraints of limited biological replication across specific experimental groups, all microbial community diversity analyses presented herein represent exploratory investigations rather than confirmatory statistical validations. These findings serve to generate testable hypotheses and provide preliminary taxonomic insights into kombucha microbiome architecture but must be interpreted with appropriate statistical caution and validated through future investigations incorporating expanded sample sizes and enhanced experimental replication design.

Importantly, amplicon based 16S rRNA gene (V3–V4 hypervariable regions) and internal transcribed spacer (ITS) sequencing approaches have well documented limitations in taxonomic resolution, particularly for phylogenetically closely related genera such as *Komagataeibacter* and *Zygosaccharomyces*. Therefore, taxonomic assignments in this study are reported predominantly at the genus level, except where sequence identity exceeded established species-level classification thresholds. Species level taxonomic designations are presented conservatively and should be interpreted as putative assignments requiring further validation through whole-genome sequencing or multilocus sequence typing approaches [28].

**Fig. 3:**
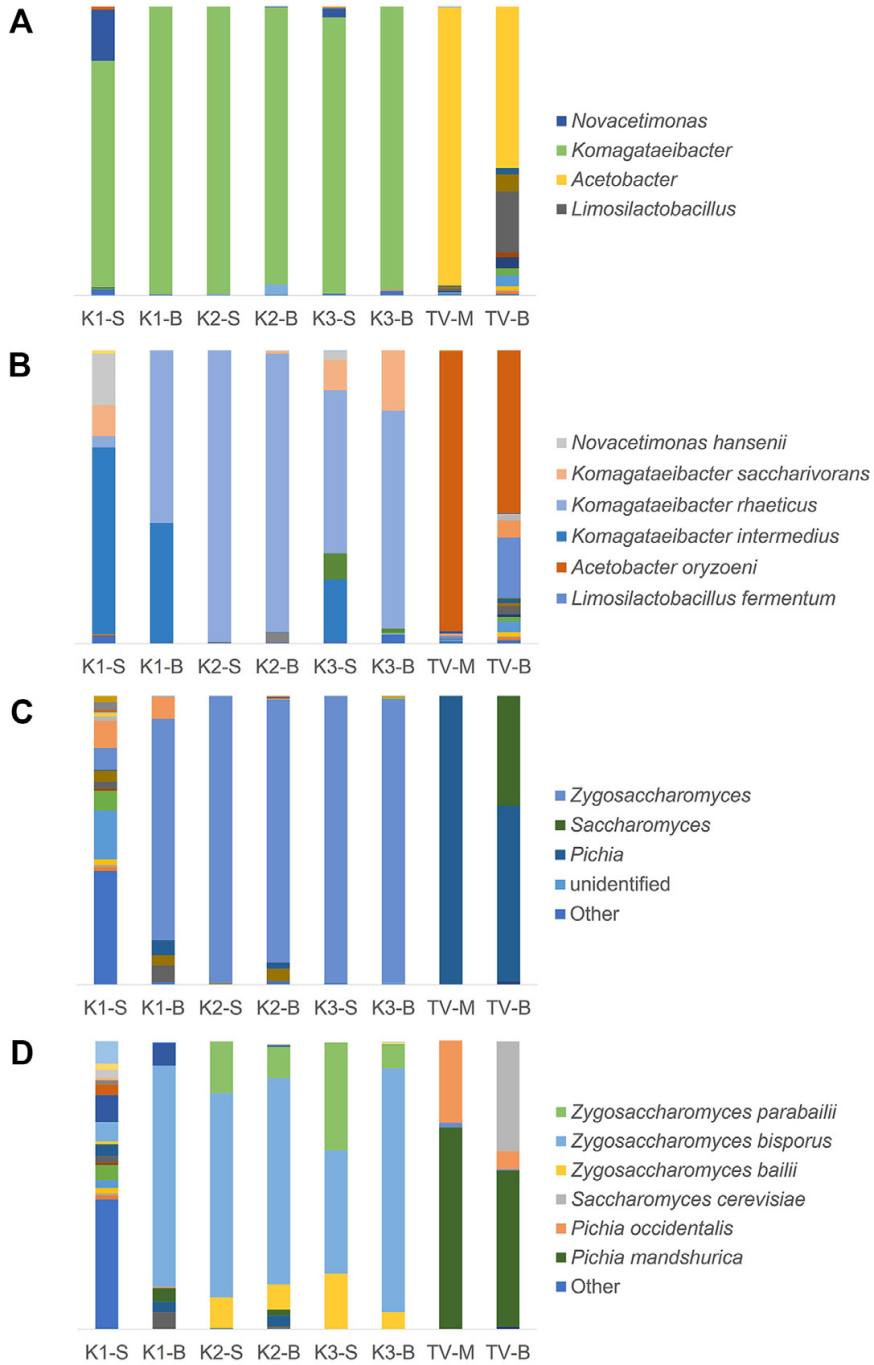
Taxonomy summary plots. Taxonomic assignments of bacterial and yeast communities are shown as bar charts. The height of the bar indicates the abundance of 16S rRNA and ITS 3–4 reads assigned to each taxon. Taxa with less than 0.2% abundance in each sample are displayed as “Other.” (**A**) Bacterial genus level, (**B**) Bacterial species level, (**C**) Yeast genus level, (**D**) Yeast species level.

In kombucha and TV samples, the predominant bacterial genera were *Komagataeibacter* and *Acetobacter*, respectively. Among the kombucha fractions, *Novacetimonas* was notably abundant in K1-S, while *Limosilactobacillus* was enriched in TV-B. At the species level, *K. rhaeticus* was the dominant taxon in all kombucha samples, except K1-S, which was primarily dominated by *K. intermedius*. In TV fractions, *Acetobacter oryzoeni* predominated in TV-M, while *Limosilactobacillus fermentum* was the second most abundant species in TV-B. These findings confirm clear distinctions in the bacterial community structure between kombucha and TV at both genus and species levels, with cellulose producing AAB prevailing in kombucha (Fig. 3A and 3B).

Regarding eukaryotic communities, K1-S exhibited a broad diversity of yeast genera, whereas the other kombucha samples predominantly showed *Zygosaccharomyces*. In contrast, *Pichia* dominated the TV samples, with *Saccharomyces* being particularly prevalent in TV-B. At the species level, K1-S harbored a wide range of yeast taxa, while other kombucha fractions were mainly dominated by *Z. bisporus* and *Z. parabailii*. TV samples showed *Pichia manshurica* as the dominant species, with *P. occidentalis* and *Saccharomyces* spp. enriched in TV-M and TV-B, respectively. Collectively, these results highlight prominent differences in eukaryotic community profiles between kombucha and TV (Fig. 3C and 3D).

**Fig. 4:**
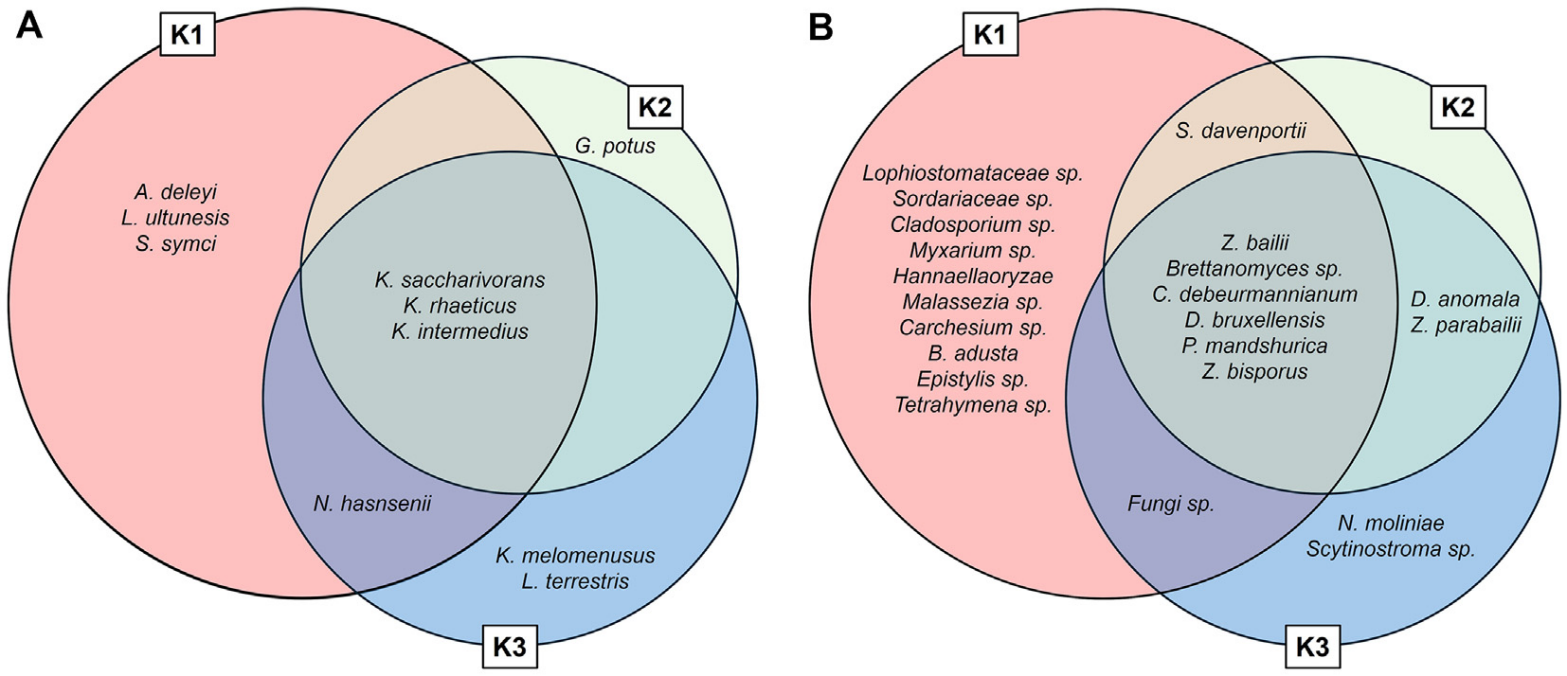
Venn diagram showing overlaps among three kombucha samples. (**A**) Bacterial species level, (**B**) Yeast species level.

To identify the core microbial taxa in kombucha samples, overlap and exclusivity analyses were performed using Venn diagrams derived from the NGS data (Fig. 4). Three bacterial species, *K. saccharivorans*, *K. rhaeticus*, and *K. intermedius*, were consistently detected across all kombucha samples and constituted the core bacterial microbiota. Similarly, the yeast species *Z. bailii*, *Brettanomyces*, *Cutaneotrichosporon debeurmannianum*, *D. bruxellensis*, *Pichia manshurica*, and *Z. bisporus* were present in all kombucha fractions. This conserved consortium, primarily comprising cellulose producing *Komagataeibacter* spp. and fermentative yeasts such as *Zygosaccharomyces* and *Brettanomyces*, is likely responsible for the unique biochemical and fermentation characteristics of kombucha.

In contrast, TV samples harbored microbial communities dominated by *Acetobacter*, *Pichia*, and *Saccharomyces*, thus showing a clear distinction from the kombucha specific core taxa. These findings suggest that while peripheral taxa may influence product specific flavor profiles and secondary metabolite production. However, the presence of the core microbiota, particularly *Komagataeibacter* and key fermentative yeasts, could serve as the defining microbial signature of kombucha. This conserved consortium may play a critical role in promoting the coordinated production of metabolites and biopolymers during fermentation.

### Changes in Major Metabolites and Fermentation Parameters during Kombucha Fermentation

**Fig. 5:**
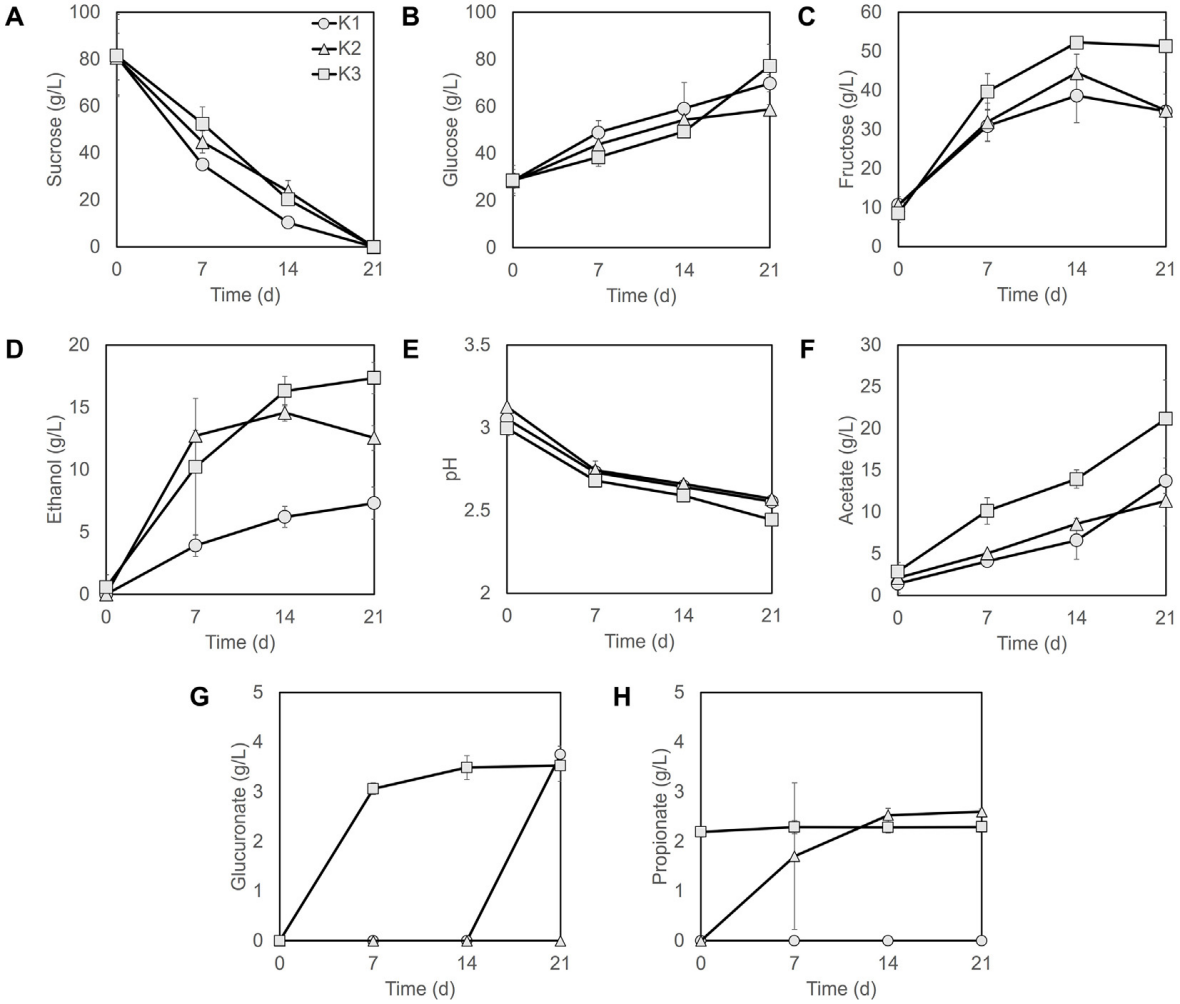
Metabolic profiling of three kombucha samples during fermentation. (**A**) Sucrose, (**B**) Glucose, (**C**) Fructose, (**D**) Ethanol, (**E**) pH, (**F**) Acetate, (**G**) Glucuronate, (**H**) Propionate. Values are presented as mean ± standard deviation (SD, *n* = 3).

The dynamics of sucrose, glucose, fructose, ethanol, acetate, glucuronate, and propionate concentrations and pH were monitored in three kombucha samples (K1–K3) over a 21 day fermentation period (Fig. 5).

Sucrose concentrations declined rapidly across all samples, and sucrose was nearly depleted by day 21 (Fig. 5A), indicating its efficient utilization by the microbial consortia. However, glucose and fructose concentrations increased during the early to mid stages of fermentation, peaking between days 14 and 21 (Fig. 5B and 5C). These trends are consistent with yeast derived invertase activity, which hydrolyzes sucrose into monosaccharides that serve as substrates for subsequent fermentation processes [29].

Ethanol concentrations increased sharply within the first 7 days, with the K3 sample showing the highest level (16.31 g/l) on day 14, followed by K2 (14.57 g/l) and K1 (6.21 g/l) (Fig. 5D). After day 14, ethanol concentrations plateaued or slightly declined, suggesting AAB mediated oxidation of ethanol into acetic acid.

Initial pH values ranged from 2.99 to 3.13 and steadily decreased to approximately 2.5 by day 21 (Fig. 5E). This acidification of the fermented sample correlated with a continuous increase in acetate concentrations throughout fermentation, indicating a direct relationship between organic acid production and pH reduction. K3 exhibited the most prominent pH decrease, which aligned with its highest level of acetate accumulation (21.15 g/l on day 21), thereby reflecting more active oxidative metabolism and enhanced potential for acid production (Fig. 5F).

Glucuronate concentrations increased over the course of fermentation, with K3 exhibiting the most consistent and highest accumulation (3.53 g/l), whereas K2 produced negligible amounts (Fig. 5G). These data support that kombucha fermentation yields physiologically relevant levels of organic acids, including glucuronate, within the range reported across kombucha systems [30]. In particular, bacterial cellulose producing acetic acid bacteria in the families *Acetobacteraceae*, notably the genera *Gluconacetobacter* and *Komagataeibacter*, are implicated in glucuronate formation via glucose dehydrogenase mediated oxidation and subsequent oxidative steps during fermentation [31, 32].

Propionate was identified as a short chain fatty acid (SCFA) produced during kombucha fermentation and was detected in both K2 and K3, with K3 maintaining relatively stable levels at 2.27 ± 0.11 g/l across the fermentation period and K2 increasing to 2.6 g/l by day 21 (Fig. 5H). These concentrations indicate that kombucha fermentation can yield physiologically meaningful amounts of SCFAs, a class of metabolites widely recognized as key components of postbiotics that contribute to antimicrobial, immunomodulatory, and barrier supporting functions [33, 34]. In line with this, NGS confirmed the presence of lactic acid bacteria (LAB) in the kombucha microbiome (Fig. S1).

Collectively, these findings delineate a tightly orchestrated metabolic cascade during kombucha fermentation, comprising sucrose hydrolysis, monosaccharide accumulation, ethanol formation, subsequent oxidation of ethanol to acetate by acetic acid bacteria, and progressive acidification of the broth [29]. Within this scheme, acetate functions both as a salient metabolic marker and as a principal determinant of the beverage’s biochemical profile and acidity, reflecting the core activity of acetic acid bacteria in kombucha ecosystems [35].

Among the organic acids generated during kombucha fermentation, glucuronic acid is particularly prominent, originating from microbial oxidation of glucose and being associated with xenobiotic detoxification and hepatoprotective effects [30, 36] In addition, its metabolic routing toward glucosamine highlights a putative role in ascorbate precursor pathways, offering a biochemical rationale for the beverage’s reported antioxidant and anti-inflammatory activities [37, 38]. Propionic acid has been reported to provide health benefits to the host [39]. In particular, it is known to reduce lipid synthesis and promote lipid oxidation. These effects may be consistent with previous findings that kombucha consumption might reduce LDL and total cholesterol levels while potentially increasing HDL concentrations in the bloodstream [40, 41]. The detection of glucuronate and propionate suggests that these bioactive metabolites may be relevant in kombucha with possible health promoting effects. Beyond physiological relevance, these metabolites may hold industrial value if quantified as quality control markers to support product consistency and reliability across production batches. Given that glucuronate accumulation is typically associated with acetic acid bacteria, whereas propionate formation is characteristic of lactic acid bacteria, rational starter selection and fermentation optimization are expected to modulate their production profiles. Although further validation is required, such process directed strategies could enhance the functional value of kombucha and provide a framework for future studies aiming to broaden its applications in health oriented foods and beverages.

### Identification and Quantification of Cellulose Production by Bacterial Strains Isolated from Commercial Kombucha

**Fig. 6:**
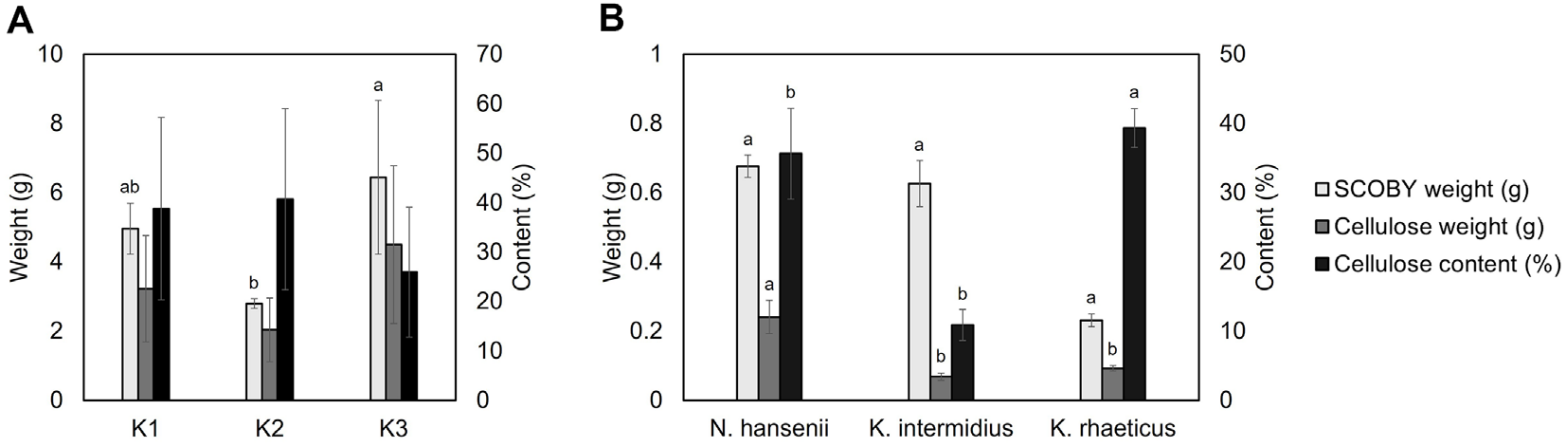
Cellulose production and content in SCOBY synthesized by bacterial strains isolated from kombucha samples. (**A**) SCOBY weight (g), cellulose weight (g), and cellulose content (%) in kombucha samples (K1, K2, and K3). (**B**) SCOBY weight (g), cellulose weight (g), and cellulose content (%) in bacterial strains isolated from kombucha samples (*N. hansenii*, *K. intermedius*, and *K. rhaeticus*). Values are presented as mean ± standard deviation (SD, *n* = 3). Statistical significance was determined using Scheffe test, and different letters indicate significant differences (*p* < 0.05).

The pellicle, which is commonly regarded as a byproduct of kombucha fermentation and known as SCOBY, contains BC with potential applications in diverse industrial and biomedical fields. Optimization of microbial community structure to enhance BC yield is proposed as a strategy to improve its utilization [42]. Despite the potential of BC for use in various applications, only a few studies have examined microbial contribution to BC productivity in kombucha. To develop strategies for enhancing BC production, cellulose content and yield were assessed in the three kombucha samples (K1–K3), along with microbial community analysis to elucidate the relationship between microbiota composition and cellulose biosynthesis (Fig. 6).

To quantify cellulose production, SCOBY weight, cellulose content, and total cellulose mass were determined (Fig. 6A). Among the samples, K3 exhibited the highest SCOBY weight (6.45 ± 2.22 g), showing a statistically significant difference compared to the other groups, whereas K2 yielded the lowest biomass (2.8 ± 0.14 g). Notably, although K3 had a relatively low cellulose content (25.95 ± 13.12%), its substantial biomass contributed to the highest total cellulose yield (4.50 ± 2.28 g).

To identify high cellulose producing strains, the cellulose production capacity of bacterial isolates obtained from kombucha samples was evaluated (Fig. 6B). *N. hansenii* showed the highest cellulose yield, producing 0.68 ± 0.03 g of SCOBY with a cellulose content of 35.66% ± 6.55%, resulting in an overall cellulose yield of 0.24 ± 0.05 g. In contrast, *K. rhaeticus* exhibited a higher cellulose content (39.35 ± 2.82%) but formed substantially less biomass (0.23 ± 0.02 g), leading to an overall cellulose yield of only 0.09 ± 0.01 g. Microbial profiling revealed that the K3 sample was predominated by *K. rhaeticus*, a known industrial BC producer [43]. The yeast community was dominated by *Z. bisporus*, which rapidly metabolizes carbohydrates into organic acids and ethanol [44].

Sucrose is not directly transported across the bacterial cytoplasmic membrane, and instead it is first hydrolyzed by periplasmic invertase into glucose and fructose, a prerequisite that can delay the onset of cellulose biosynthesis [45]. Yeast derived invertase accelerates sucrose hydrolysis, thereby increasing the pool of monosaccharides that acetic acid bacteria can directly assimilate to support growth and bacterial cellulose (BC) biosynthesis [46]. The resulting organic acids and ethanol modulate pH and nutrient availability, while AAB contribute protective biofilms. Together, these factors stabilize the consortium and limit invasion by exogenous microbes [47]. Together, these complementary interactions provide a mechanistic basis for the elevated cellulose yields frequently observed in yeast bacterium co-cultures. These metabolic byproducts possibly created a favorable environment for AAB by modulating pH and nutrient availability, thereby supporting bacterial survival and enhancing cellulose production [48]. Among the yeast taxa, *Z. bisporus* was particularly abundant. This species can synergistically enhance microbial cellulose biosynthesis when co-cultured with cellulose producing bacteria, thereby achieving high production yields [49].

Collectively, these findings confirm that the K3 kombucha sample possessed superior cellulose producing capacity, highlighting the critical role of specific bacterial yeast interactions in promoting BC biosynthesis. These results further suggest that kombucha fermentation systems can be utilized not only for beverage production but also as a platform for producing cellulose based biomaterials.

## Conclusion

This study delivers an integrated characterization of microbial communities in commercially available kombucha products by combining culture based isolation with next-generation sequencing (NGS) based diversity profiling, thereby enabling robust cross validation of taxonomic assignments and strain recovery. *Komagataeibacter* was identified as the predominant bacterial genus, with *K. rhaeticus*, *K. intermedius*, and *N. hansenii* serving as key cellulose producers. Yeast populations were dominated by *Z. bisporus* and *Z. parabailii*. Distinct compositional differences were evident between SCOBY associated and broth associated microbiota.

Critically, this study quantitatively linked isolated strains to BC output, advancing beyond previous kombucha microbiome surveys that focused primarily on community profiling without functional readouts. The highest BC yield was observed in the K3 sample, which was dominated by *K. rhaeticus* and *Z. bisporus*, consistent with synergistic interactions between acetic acid bacteria and fermentative yeasts during cellulose biosynthesis.

Collectively, the findings define a stable core microbiota in kombucha and provide quantitative evidence connecting microbial taxonomy to BC productivity, establishing a framework for designing controlled consortia that optimizes beverage quality and cellulose-based biomaterial manufacturing at an industrial scale.

## Supplemental Materials

Supplementary data for this paper are available on-line only at http://jmb.or.kr.



## Figures and Tables

**Table 1:** List of bacteria and yeast isolated from kombucha and TV samples.

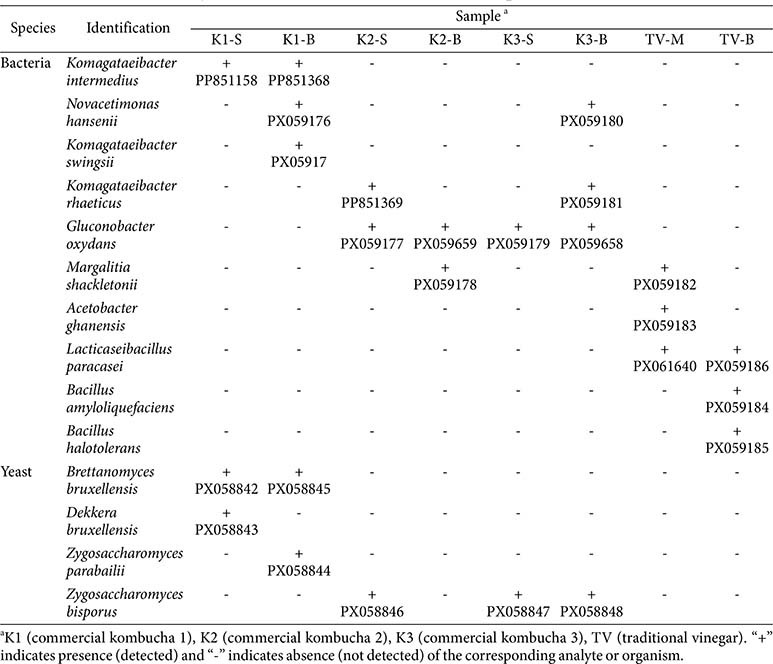

**Table 2:** Results of sequencing and diversity analysis of bacterial and yeast communities from kombucha and TV samples.