# Supplementary material for: Comparative Analysis of Microbial Communities and Biopolymer Production in Kombucha
Source: J Microbiol Biotechnol. 2025 Oct 28;35:e2508004. doi: 10.4014/jmb.2508.08004 (PMC12602875; doi:10.4014/jmb.2508.08004)
Supplement: Supplementary file 1 [file jmb-35-e2508004-supple.pdf]

J. Microbiol. Biotechnol. 2025. 35: e2508004

https://doi.org/10.4014/jmb.2508.08004

Supplementary Figure

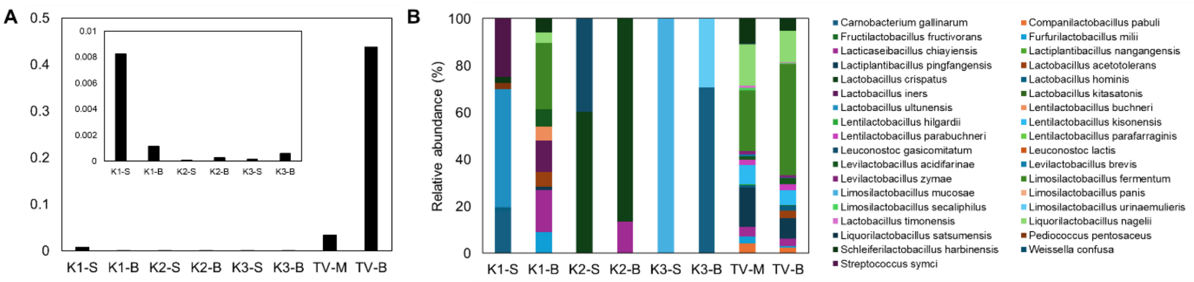

Fig. S1. Taxonomic profiling of lactic acid bacteria (LAB) at the bacterial species level. **(A)** Number of LAB species identified per sample by 16S rRNA sequencing. **(B)** Species-level taxonomic composition of LAB communities displayed as 100% stacked bar charts.
